# Analysis of the uncharted, druglike property space by self-organizing maps

**DOI:** 10.1007/s11030-021-10343-y

**Published:** 2021-10-28

**Authors:** Gergely Takács, Márk Sándor, Zoltán Szalai, Róbert Kiss, György T. Balogh

**Affiliations:** 1 Department of Chemical and Environmental Process Engineering, Budapest University of Technology and Economics, Műegyetem rakpart 3, Budapest, 1111 Hungary; 2 Mcule.com Kft, Bartók Béla út 105-113, Budapest, 1115 Hungary; 3 Department of Pharmacodynamics and Biopharmacy, University of Szeged, Szeged, 6720 Hungary

**Keywords:** Self-organizing maps, Compound libraries, Physicochemical properties, Chemical space, Druglikeness

## Abstract

Physicochemical properties are fundamental to predict the pharmacokinetic and pharmacodynamic behavior of drug candidates. Easily calculated descriptors such as molecular weight and logP have been found to correlate with the success rate of clinical trials. These properties have been previously shown to highlight a sweet-spot in the chemical space associated with favorable pharmacokinetics, which is superior against other regions during hit identification and optimization. In this study, we applied self-organizing maps (SOMs) trained on sixteen calculated properties of a subset of known drugs for the analysis of commercially available compound databases, as well as public biological and chemical databases frequently used for drug discovery. Interestingly, several regions of the property space have been identified that are highly overrepresented by commercially available chemical libraries, while we found almost completely unoccupied regions of the maps (commercially neglected chemical space resembling the properties of known drugs). Moreover, these underrepresented portions of the chemical space are compatible with most rigorous property filters applied by the pharma industry in medicinal chemistry optimization programs. Our results suggest that SOMs may be directly utilized in the strategy of library design for drug discovery to sample previously unexplored parts of the chemical space to aim at yet-undruggable targets.

**Graphic abstract:**

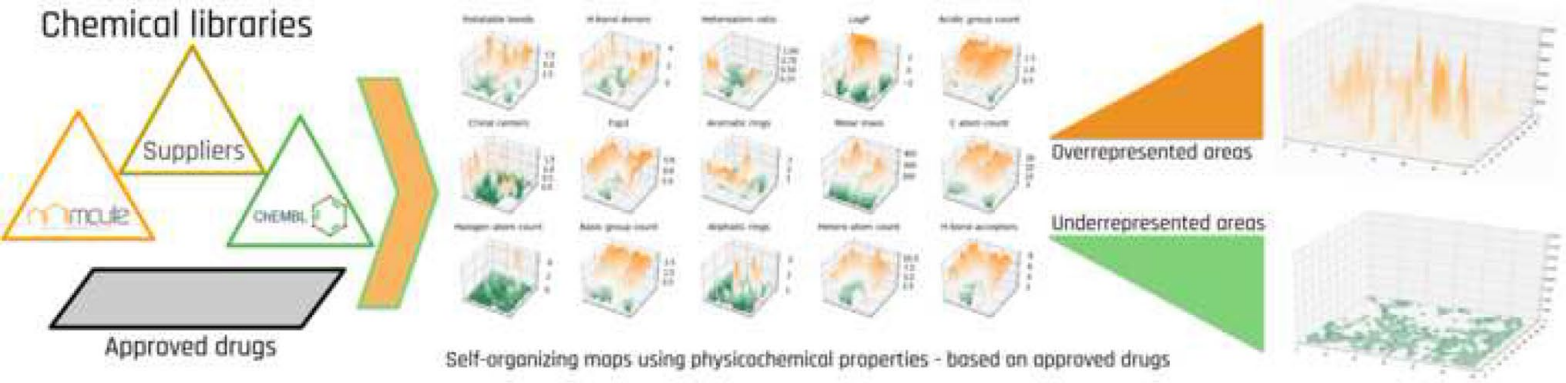

**Supplementary Information:**

The online version contains supplementary material available at 10.1007/s11030-021-10343-y.

## Introduction

The selection of molecules based on calculated properties (a.k.a. virtual screening) is a fundamental approach which is widely applied at various stages of drug discovery to reduce its costs and time consumption. Such methods run calculations/simulations on a digital representation of a molecule vs. running real experiments on the physical sample. A large number of molecular representations/descriptors have been reported that capture some key features of the molecules that may be relevant at different stages of the drug discovery process [1–9] These descriptors widely vary in their accuracy (correlation with experimental results), and their prediction method in terms of cost and time demands.

Most drug targets have some unique characteristics that can be used to define the structural/property space of compounds with selective inhibitory/agonist effects. On the other hands, orally administered drugs typically share some common properties that correlate with a desirable ADMET profile. Physicochemical properties are of outstanding importance as they have been shown to highly correlate with the pharmacokinetic (PK) profile of drug and drug-like compounds. In their landmark paper, Lipinski et al. [1] showed that a few simple rules based on physicochemical properties (rule-of-five, Ro5) can effectively select compounds for drug availability, especially in terms of PK; thus, the concept of druglikeness has been born. Since most of these properties can be calculated very cost- and time-efficiently, they can be easily integrated into any drug discovery workflow aiming for an orally available drug. Since the publication of Ro5 many similar rules and algorithm has been developed to further improve such property-based filters focusing on different approaches (e.g., fragments) [10], stages of development (e.g., lead-likeness) [11, 12] and the physiological compartment of the target (e.g., MPO for CNS drug development) [13]. While the effect of individual physicochemical properties and their linear combinations on the PK profile of molecules have been extensively studied, there has been less published analysis on more complex property profiles. For example, Ro5, Ro3 [10] and Pfizer’s CNS MPO [14], three widely used parameters derived from physicochemical properties assume that the desirability of the individual properties does not depend on each other. In other words, the desirable and undesirable property ranges are defined individually, and thus, they are independent. As a consequence, two unfavorable properties can never have a favorable contribution to the Ro5, Ro3 or MPO combined parameters. As an example, if compound “A” has unfavorable lipophilicity (logP > 5) and compound “B” has an unfavorable number of H-bond acceptors (HBA > 10), compound C with logP > 5 and HBA > 10 will be—by definition—unfavorable. However, there might be certain property combinations that do not pass Ro5 but are still favored and thus they are worth to explore. In fact, there is a significant number of approved, orally administered drugs which are beyond Ro5. Moreover, there are several explanations for physicochemical properties that allow these drugs to compensate for their unfavorable properties including the formation of intramolecular hydrogen bonding and macrocyclization [15], 16. Consequently, for the analysis of drugs in property space, it is more adequate to apply approaches and methods that can effectively capture unusual, but favorable property combinations too [17]. This could potentially lead to the discovery of novel ligands against difficult targets currently considered as undruggable [18–20].

Some methods that are capable of identifying complex property profiles include self-organizing maps (SOM) [21–23], principal component analysis (PCA) [24], generative topographical mapping (GTM) [25] stochastic neighbor embedding (SNE) [26], etc.

All of these methods have been already applied in some context to chemical space navigation/chemography; however, the majority of these studies have been focusing on structural patterns and motifs.

Since physicochemical properties are fundamental in predicting pharmacological properties and are widely used for classification and filtering datasets, our aim was to carry out a comprehensive analysis and examine the purchasable compound libraries in the physicochemical space and check if there are significant underrepresented/overrepresented regions. As a common and easily interpretable dimension reduction algorithm, we have chosen to use self-organizing maps. SOMs are classified as machine learning algorithms. They were introduced by Kohonen et al. [21] and since then they have been applied for chemical space navigation, discrimination of libraries for various targets based on a variety of structural elements or properties [27–34] It is a widely known method that is easily interpretable and visualizable. To the best of our knowledge, however, SOMs have not been applied for the analysis and comparison of various supplier catalogs and thereby for the exploration and exploitation of currently unpopulated parts of the purchasable and druglike chemical space using physicochemical properties as descriptors. The approach of SOM is completely transparent compared to deep neural networks in the sense that no hidden layers or various weighting methods are applied. Consequently, they can be easily applied for data representation and visualization. Initially, random property values for all analyzed properties are assigned to each point (neuron) of the 2-dimensional map. In each iteration, one element of the training dataset is paired to the neuron with the lowest Euclidian distance from the current element, and the paired neuron and its neighborhood are adjusted to become more similar to that element (learning). The pairing and learning steps are executed multiple times for the training set, while both the learning rate (modifying coefficient) and the learning distance (size of the neighborhood) are gradually decreased. In the resulting map, the elements of the training set are homogeneously distributed. Any new datasets to be analyzed can be placed onto the map, where each element of the dataset will be assigned to the most similar point of the map (i.e., the point with the lowest Euclidian distance from the element). Some of the key advantages of SOMs include that they can be applied to a large number of variables, and they represent the physicochemical property space as a continuous, easily interpretable and intuitive map without any data loss. These characteristics made it the method of choice for our purposes compared to PCA, GTM or SNE. Nevertheless, some more complex models and algorithms may also be suitable for our goals, but they would come at a cost of losing some level of interpretability and transparency—in comparison to self-organizing maps.

In SOM, the neurons of the maps represent complex property profiles unlike the simple linear combinations such as Ro5, and thus, they should be able to identify unusual but druglike physicochemical property concurrences that might enable the targeting of yet-undruggable sites. For example, there are a lot of allosteric sites on macromolecules that are yet unexplored, and it has been shown that such sites accommodate molecules with different pharmacokinetic profiles compared to those binding to orthosteric sites. In particular, allosteric ligands represent one of the most promising strategies to target kinases and GPCRs selectively [35]^36^. The shortages of chemical space characterization by MPO, Ro5 and similar approaches are reflected by several known drugs with unusual and complex property profiles filtered out by such filters [37]^1516^.

Numerous articles have been published in the last decades that aimed to develop a method that is capable to navigate in chemical space and identify unpopulated parts. The majority of these studies applied novel or enhanced algorithms and presented a few representative examples of such underrepresented or very unique chemical space areas. [38–40] For example, Zabolotna et al. concluded that a few structural elements are common in the overpopulated parts (e.g., amides and sulfonamides) but found no difference between tangible and in-stock libraries [41]. The studies used different databases (ChEMBL, PubChem [42]) and compared different even more libraries (ChEMBL, PubChem, GDB [43], etc.); thus, they are difficult to compare. Nevertheless, their findings suggest that the analyzed databases represent a significant heterogeneity that could be potentially exploited in ligand design.

In this study, we analyzed two main types of compound sources in physicochemical property space: (i) compounds with reported bioactivity data primarily generated during drug discovery and (ii) commercially available compounds primarily designed for drug discovery. In particular, we chose sixteen physicochemical properties widely applied for library filtering by the pharmaceutical industry. SOMs were developed to homogeneously represent approved drugs in this property space. The analysis of the distributions led to the identification of underrepresented regions that resemble the property profile of approved drugs that were rigorously filtered by and thus compatible with Ro5 and further medicinal chemistry filters. Our findings reveal that a significant portion of the chemical space that is compatible with all rigorous property filters is still underrepresented by commercially available libraries. The developed SOMs can be directly utilized in the chemical library design for drug discovery, and thus, they provide a useful tool for research groups as well as chemical suppliers to exploit unidentified but potentially drug-like chemical spaces.

## Results and discussion

There is an ongoing debate on whether Ro5 or similar physicochemical property-based parameters should be applied and at which stage of drug discovery. On one hand, such filters can be extremely useful to minimize PK and toxicity issues during development, and thus, in case, there are a sufficient number of hits/leads it is suggested to prioritize them by such criteria. On the other hand, since pharmacodynamics (PD) behavior cannot be adequately described by just a few simple rules of thumbs, the application of such filters inevitably results in false negatives and false positives. While the false negatives are typically not considered as a major drawback of the application of Ro5, false positives are frequently interpreted as lost opportunities by opponents.

In our study, we analyzed the distribution of the following four chemical databases in the multidimensional property space of sixteen physicochemical properties using SOMs: the bioactivity database of ChEMBL [44] (version 23), the stock (Supplier #1 stock) and virtual (Supplier #2 virtual) databases of one of the largest chemical suppliers and the stock database of a chemical marketplace that integrates multiple supplier catalogs (Mcule stock [45]). We used the DrugBank [46] database of approved drugs as a reference to identify under- and overrepresented regions of the chemical space. First, we filtered all databases by Ro5 (Table S1) as well as by a more rigorous property filtering workflow (referred as Strict filter (Table S1) below) developed based on the instructions of large pharmaceutical companies. The primary aim of the Strict filter was to minimize compounds with any unsuitable properties or liabilities that can lead to issues during optimization.

**Fig. 1:**
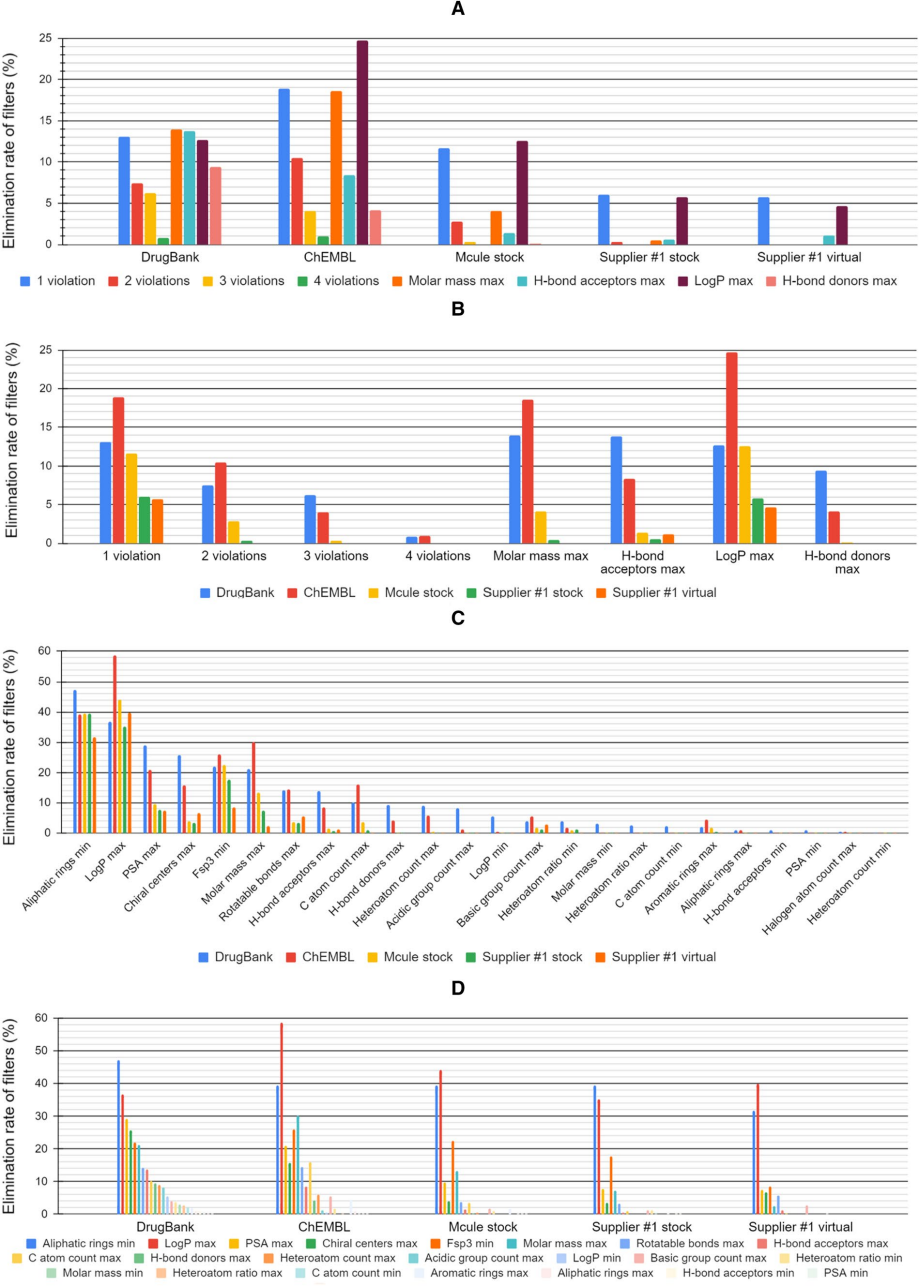
Elimination rates of the individual filters of Ro5 and the different numbers of violations (**a**, **b**) and the elimination rates of the applied Strict filters (**c**, **d**) on the analyzed databases. Exact numbers can be found in Supporting Information (Table S2)

Although a significant portion of approved drugs (1248 with min two violations and 2377 with min one violation) is filtered out by Ro5 (Table 1), the majority of them are not orally administered drugs, and therefore, they are not considered as false positives. The ratio of filtered compounds is even larger in case of the Strict filter (Fig. 1c and d). Apparently, this filter has a lot of false positives (only 9.7% of approved drugs survived the filtering). Nevertheless, the aim of such a filter is to minimize the vast number of purchasable compounds to be acquired by a pharma company to the most valuable ones with minimized risk of an undesired ADMET profile. Altogether, 90% of approved drugs failed on at least one of the Strict filter rules. Interestingly, while the elimination rate by Strict filter in DrugBank and ChEMBL was relatively high (90% and 86%, respectively), a significantly smaller portion of the Supplier #1 stock (66%), Supplier #1 virtual (66%) and Mcule stock (71%) were discarded suggesting the adaptation of supplier catalogs to the requirements of the pharma industry.

**Table 1:** Size of the unfiltered and filtered databases by Ro5, Strict property filter, substructural SMARTS and Lilly filters and their combination

	DrugBank	ChEMBL	Mcule stock	Supplier #1 stock	Supplier #1 virtual
Original size	8646	1,727,112	9,169,172	3,397,680	336,985,480
Ro5 filtered size (0 or 1 violation) (% of original)	7398 (86%)	1,459,467 (85%)	8,885,121 (97%)	3,385,630 (100%)	336,858,162 (100%)
Strict property filtered size (% of original)	836 (9.7%)	242,764 (14%)	2,669,910 (29%)	1,160,797 (34%)	115,096,630 (34%)
Substructure filtered size (% of original)	4583 (53%)	875,029 (51%)	6,755,581 (74%)	2,640,763 (78%)	287,563,008 (85%)
Strict property + Substructure filtered size (% of original)	617 (7.1%)	153,389 (8.9%)	2,332,297 (25%)	1,007,186 (30%)	103,135,958 (31%)

In fact, supplier catalogs are frequently filtered against criteria such as molar mass, polar surface area (PSA), H-bond acceptors (HBA), and H-bond donors (HBD). Consequently, there is a significantly lower elimination rate of these filters in the Supplier #1 stock, Supplier #1 virtual and Mcule stock libraries. The four property filters that eliminated the largest portion of approved drugs are as follows (elimination rates in %): minimum number of aliphatic rings (47%), maximum of logP (37%), maximum of PSA (29%) and maximum number of chiral centers (26%). Interestingly, the aliphatic ring filter showed a significantly larger elimination rate of approved drugs vs. other libraries suggesting that although there is a trend for a higher preference for aliphatic vs. aromatic rings by the pharma industry, this is not a requirement for drug approval. Nevertheless, planar (aromatic, low fsp3) compounds tend to aggregate and to be identified as false positives in, e.g., cell-based screening assays, and thus, their exclusion is justified by the associated higher risk [47]. Similarly, the higher elimination rate of the maximum number of chiral centers filter in the approved drug library indicates that higher number of chiral centers does not imply a higher chance for failure in clinical trials, but the separation of the different stereoisomers makes the medicinal chemistry optimization more difficult. The strongest three filters of the commercial libraries and their elimination rates are as follows: minimum number of aliphatic rings (Supplier #1 stock: 39%, Supplier #1 virtual: 32%, Mcule stock: 39%), maximum of logP (Supplier #1 stock: 35%, Supplier #1 virtual: 40%, Mcule stock: 44%) and the minimum fraction of sp3 carbon atoms (Supplier #1 stock: 18%, Supplier #1 virtual: 8%, Mcule stock: 23%). The high numbers for the aliphatic ring and fsp3 filters suggest that the industry is still in the process to adapt to the recent trend of favoring compounds with more pronounced 3D characteristics. Nevertheless, the high elimination rate of logP is surprising after the decades of the publication of Ro5 and several other studies [48]^49^ highlighting the correlation of high lipophilicity with promiscuity (unspecific binding) and toxicity issues. It has to be mentioned that the absolute value of the elimination rate of max. logP was the highest in ChEMBL (59%) compared to the other libraries (Supplier #1 stock (35%), Supplier #1 virtual (40%), Mcule stock (44%) and DrugBank (37%)) (Fig. 1c). A similar trend was observed for the logP filter in case of Ro5 (Fig. 1a). This suggests that approved drugs indeed have a sweet-spot for logP, while ChEMBL database contains a higher number of “tool” compounds with a less favorable PK profile but still sufficient for target validation.

It is important to mention that the commercially available compounds have not been exclusively designed for drug discovery and thus can serve multiple purposes. Nevertheless, drug discovery represents currently the largest market for chemical libraries, and thus, it is the main driving force of the chemical library provider industry. Another important factor that may have affected our results and analysis is that there is no standard calculation of certain properties that may partially explain, e.g., the large number of compounds discarded due to high logP.

After the analysis of the property filters, our primary goal was to investigate whether the chemical space compatible with the Strict filter (i.e., interest of the pharma industry) still contains significant unexploited regions. If it does, such regions are of high potential as they represent compounds with a minimal chance for undesired pharmacokinetics, and thus, they should be pursued with higher priority over-filtered out ones. If it does not, the results suggest that strict property filters may need to be softened in order to improve the sampling of unexplored portions of the property space. It is important to mention that the chemical space filtered out by Strict filter should still contain numerous potential drug candidates; however, they have a higher risk of failure in medicinal chemistry optimization. Consequently, the intention of our analysis was not to cover the complete chemical space of drugs, but to focus on a subset with the lowest risk, i.e., to minimize false positives.

We trained a self-organizing map (SOM) using the properties of approved drugs filtered by Strict filter. This resulted in a map where the drugs were homogeneously distributed, and their calculated physicochemical properties formed cliffs and valleys (see the distribution of each property in Figure S1. in Supporting Information).

**Fig. 2:**
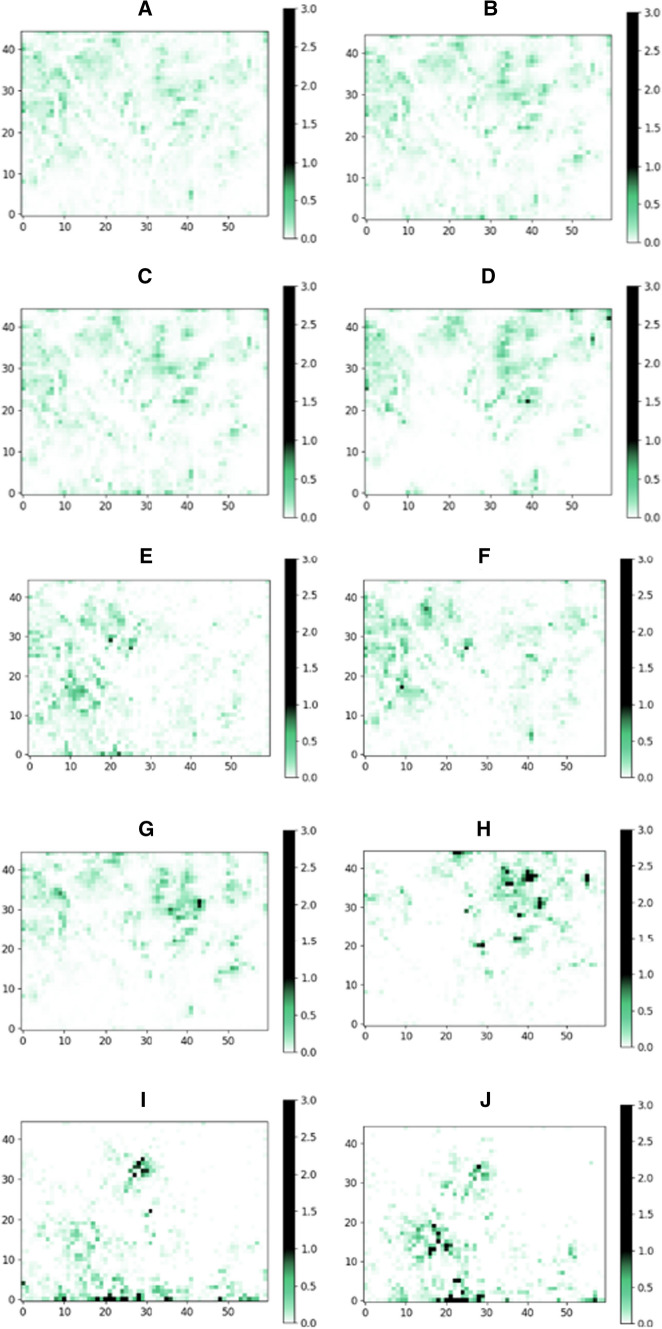
ChEMBL database (**a**), the chemical marketplace Mcule stock library (**b**) Supplier #1 stock library (**c**), and the Supplier #1 virtual library (**d**), Supplier #2 (**e**), Supplier #3 (**f**), Supplier #4 (**g**), Supplier #5 (**h**), Supplier #6 (**i**) and Supplier #7 (**j**) placed on a self-organizing map trained on approved drugs filtered by Strict filter. The values (therefore colors) of each grid points are the percent of molecules of the given database that are assigned to that point based on their properties. Maximum values in all heatmaps were set to 3.0 which is the highest value occurred in either of the databases. The green parts of the colorbars and the maps indicate the “ideal” number of molecules falling to one point—in a case of a completely heterogenic database

Ten libraries were chosen for this analysis: six supplier’s stock catalogs and one supplier’s virtual catalog, the database of Mcule (chemical marketplace) and ChEMBL (bioactivity database). Each library was filtered by Strict filter, and the remaining compounds were placed onto the SOM trained by the approved drugs set prefiltered by Strict filter. In case of the ideal sampling of the property space, we expect a homogenous distribution of the molecules. The distributions, however, were found to be surprisingly heterogeneous: The majority of the maps were largely underrepresented (white) and some largely overrepresented regions (black, dark green) could be identified (Fig. 2). These results suggest that there are many, druglike property profiles that are currently underrepresented in the purchasable chemical space. These yet-unexplored regions might contain ligands for those targets that require novel and uncommon approaches to target. Such regions of the chemical space comply with all the strictest pharma industry criteria and therefore represent an opportunity for drug discovery. The individual distributions of supplier catalogs showed heterogeneity to a different extent. We introduced map heterogeneity score to characterize and quantify the heterogeneity of the individual databases and their regions calculated as the number of largely overrepresented (average number of molecules per grid point *5) and largely underrepresented (average number of molecules per grid point / 5) regions (grid points) divided by the number of all grid points (Table 2).

**Table 2:** Map heterogeneity scores of different supplier libraries and the number of molecules at overrepresented grid points of the map

	Suppliers									
	ChEMBL	Mcule stock	#1 stock	#2 stock	#1 virtual	#3 stock	#4 stock	#5 stock	#6 stock	#7 stock
Total molecules	242,765	2,669,910	1,160,797	11,695	110,507,688	18,145	164,288	10,034	5,793	3,965
Map heterogeneity score	0.31	0.39	0.40	0.44	0.54	0.54	0.56	0.67	0.74	0.81
Molecules in overrepresented regions (% of total)	40,983 (17%)	596,227 (22%)	270,049 (23%)	4,268 (36%)	37,896,339 (34%)	6,708 (37%)	67,613 (41%)	6,175 (62%)	3,835 (66%)	2,752 (69%)

**Fig. 3:**
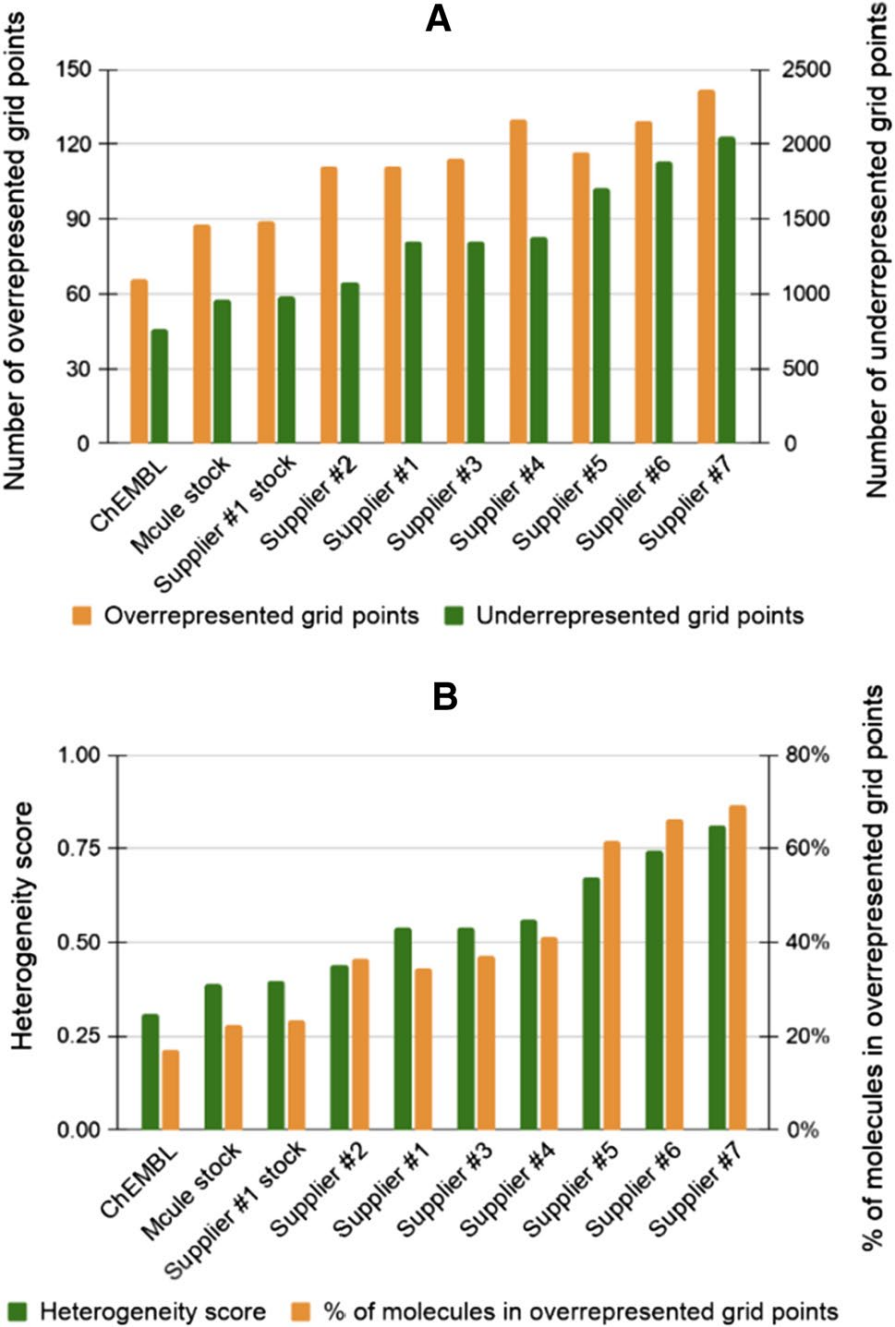
Number of overrepresented and underrepresented grid points of various libraries placed on the map (**a**) and map heterogeneity scores and the percent of libraries in overrepresented grid points (**b**)

The most homogenous distribution (representing the most heterogenous library) was observed for ChEMBL, a collection of compounds described in scientific publications generated by a large number of independent research groups worldwide. This was also reflected in the lowest map heterogeneity score. In case of ChEMBL, only 66 grid points were overrepresented containing only 15% of the molecules of the whole database. Furthermore, we identified only 766 grid points (containing 28% of the molecules) as underrepresented. ChEMBL was followed by the commercial chemical marketplace database (Mcule stock) in line with expectations that the combination of supplier catalogs yields a more diverse representation of the chemical space compared to single supplier catalogs (Fig. 3). The number of drug molecules residing in these regions represent 23% (195) of all drugs used to build the corresponding SOM.

The least diverse libraries were identified among the suppliers’ individual catalogs, which may be reasoned by the fact that suppliers typically synthesize several analogs for the same scaffold for individual projects; thus, such compounds may be less diverse compared to, e.g., ChEMBL. Furthermore, suppliers are working under time pressure and thus fewer step, robust reactions, cost-effective synthesis and availability of stock building blocks are critical for them to fulfill orders on time. On the other hands, these factors may have a negative impact on the in-house chemical diversity [50]. Interestingly, the analyzed virtual catalog of Supplier #1 showed less diversity and a more heterogeneous distribution over the map with highly overrepresented regions compared to the stock catalog of Supplier #1. This may be reasoned by the fact that while such enumerated virtual libraries can result in magnitudes higher number of potentially synthesizable molecules, they are based on a limited set of building blocks and reaction rules. Thus, their diversity is limited at least when compared to the same supplier’s stock catalog. Nevertheless, the analyzed virtual library of Supplier #1 was still more diverse than the majority of the other supplier’s analyzed stock libraries. Interestingly, we could not find a correlation between diversity and the size of the chemical libraries (Table 2).

These results suggest that the combination of a chemical marketplace and the concept of virtual libraries (i.e., a combination of diverse stock building blocks and robust chemical reactions) could yield a very large and diverse database and thus better coverage of the druglike chemical space.

**Fig. 4:**
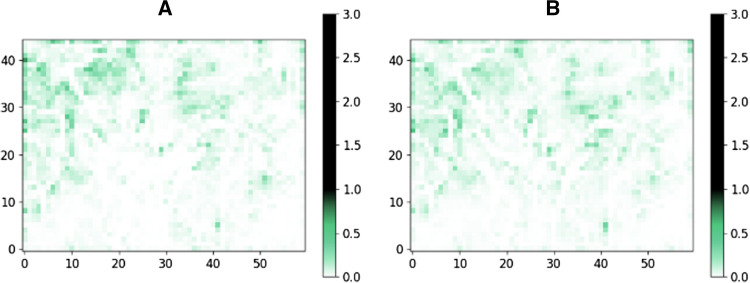
Distributions of molecules that failed the unwanted substructural filters (**a**) and the full ChEMBL database (**b**) (identical to Fig. 2a)

Since the results indicated large unexplored property regions, we wanted to confirm whether they represent real opportunities and they do not correspond to non-druglike property profiles from another perspective. For example, the presence of liable substructures can increase the chances of false positives and/or toxicity and such residues may not be identified by Strict filter. In fact, in a typical workflow of library design, physicochemical property filters are applied in combination with SMARTS-based substructural filters. We therefore postfiltered the approved drug set already processed by Strict filter against a set of unwanted substructures described in the literature (referred as SMARTS and Lilly filters) [51–53] The distribution of the underrepresented regions was compared in the presence and absence of such postfiltering of the unwanted substructures on the ChEMBL dataset. (Fig. 4).

**Fig. 5:**
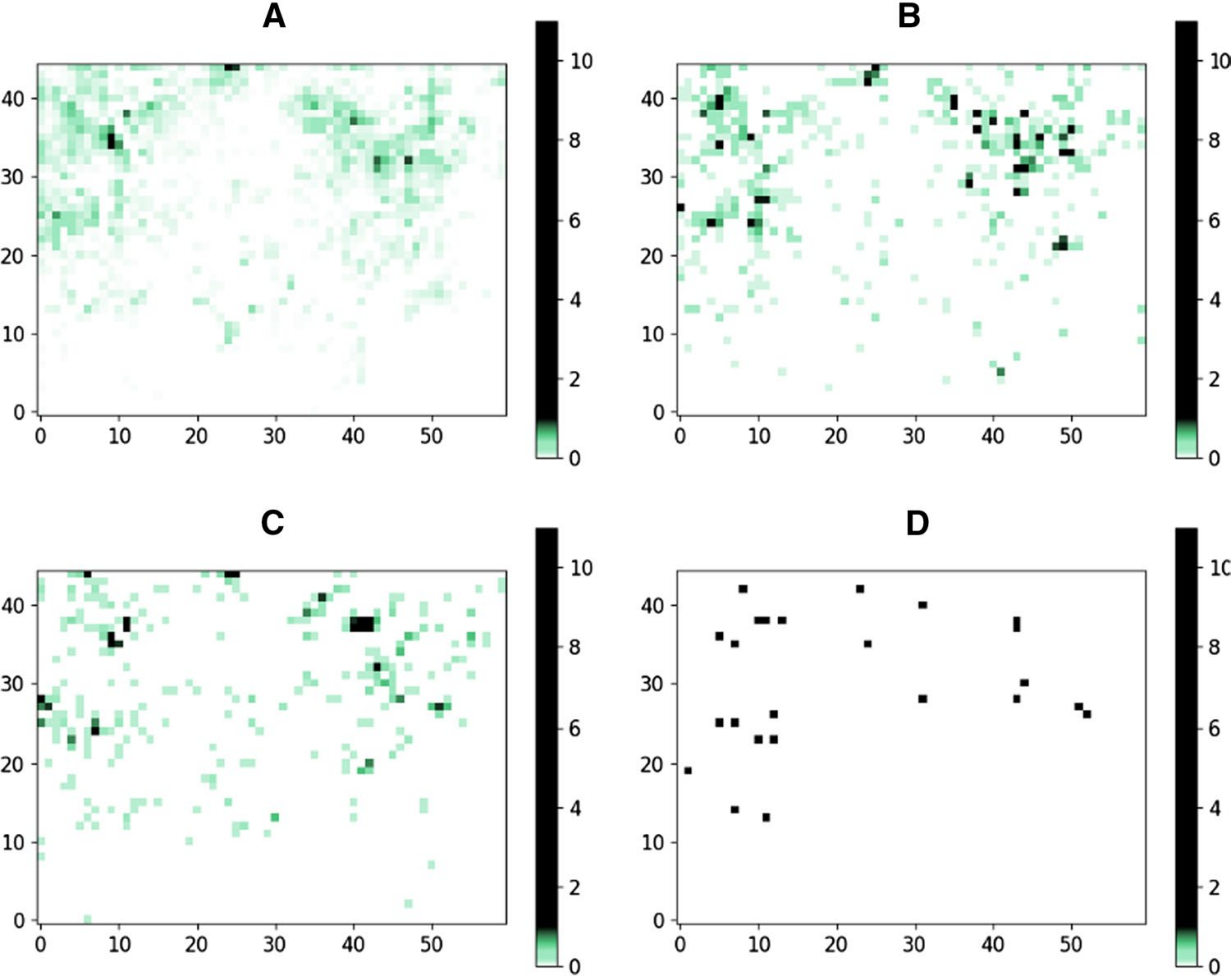
Distributions of selective (1 target) (**a**), fairly selective (targets = 2) (**b**), non-selective (2 < targets) (**c**) and particularly promiscuous (10 < targets) (**d**) compounds

In fact, a positive correlation could be identified between the overrepresented regions and the regions enriched by unwanted substructures suggesting that the underrepresented regions are not enriched in compounds containing residues of increased risk. We have compared the total number of molecules and the number of those that contained unwanted substructures. We have found that their ratio was higher than the expected value of the given grid points in 332 (covering 2586 molecules) underrepresented and 26 overrepresented (covering 16,153 molecules) points. The ratio of under/overrepresented grid points was 11.6:1 that elevated slightly to 12.8:1—not a significant difference but substructural filters must be applied with additional care. Another analysis was also carried out to further confirm the druglikeness of the identified underrepresented regions of property space. As described above, ChEMBL showed the most homogenous distribution of compounds on the map trained on approved drugs. ChEMBL is a bioactivity database containing valuable experimental data and information on which compounds were identified as active or inactive by experimental biological screening against macromolecular targets. We therefore extracted compounds from the ChEMBL database reported as active (pChEMBL > 6) on maximum 1 (i), maximum 2 (ii), or minimum 10 (iii) unique and different targets (multiple activities on the same target considered as one activity). In case, the underrepresented regions of the maps were featuring non-selective, promiscuous compounds we would expect more examples of group (iii) than groups (i) or (ii) on the underrepresented regions. We have compared the distribution of the full ChEMBL database (see Fig. 2a) with the distribution of the promiscuous molecules (Fig. 5).

While only one promiscuous grid point was identified as an underrepresented grid point containing 16 molecules (0.37% of all the ChEMBL molecules in underrepresented areas), we found three promiscuous grid points which were also identified as overrepresented corresponding to 2,156 molecules (5.15% of all ChEMBL molecules in overrepresented areas). From another perspective, the ratio of over- and underrepresented grid points of the total ChEMBL database was 66:766, while the ratio of over- and underrepresented grid points of promiscuous ChEMBL molecules was 3:1. In summary, an opposite trend (enrichment of selective ligands) was found suggesting that the underrepresented areas could be indeed populated by druglike compounds, and thus, they are of great potential.

**Fig. 6:**
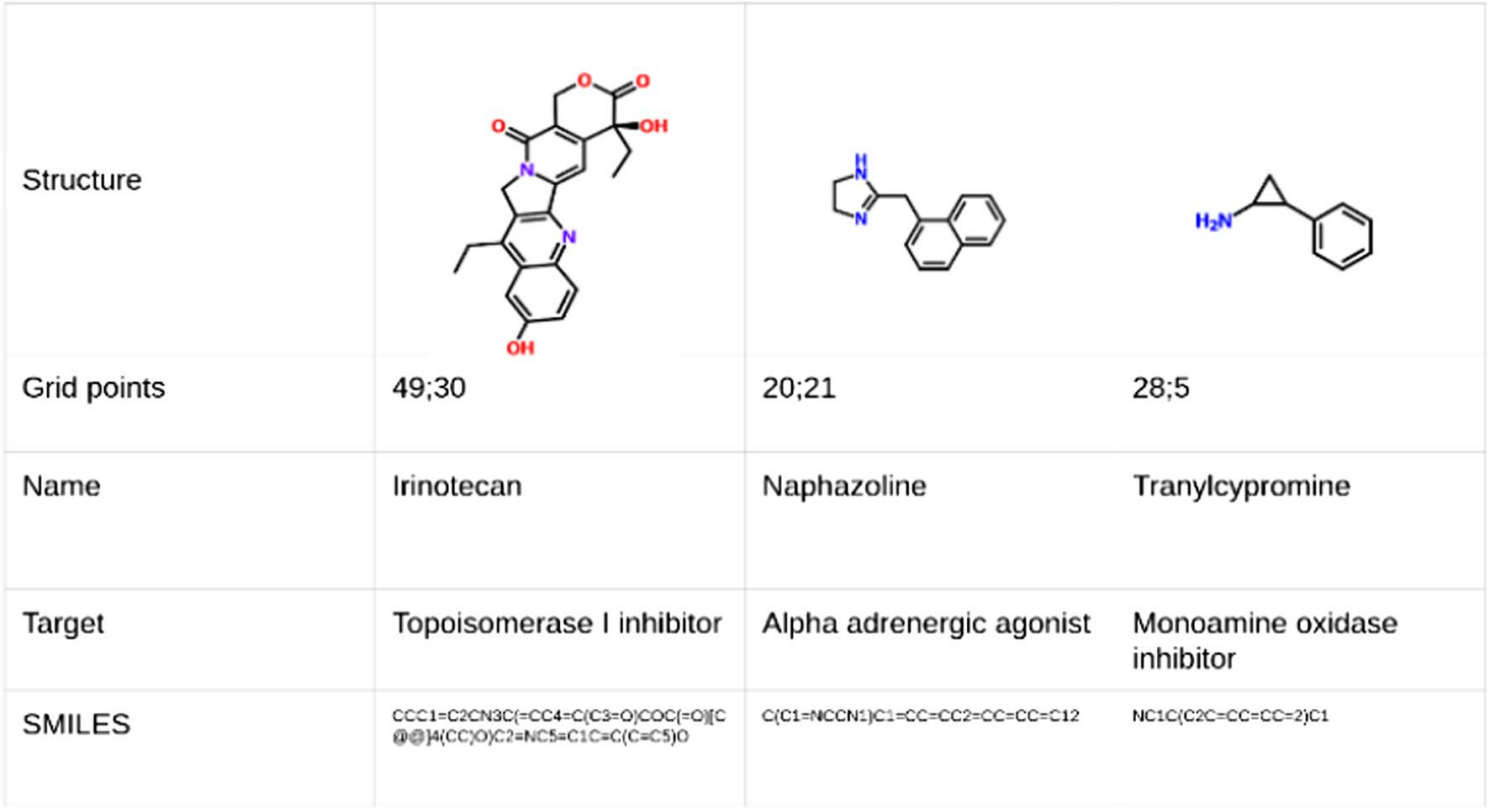
Examples of approved drugs with underrepresented property profiles

**Fig. 7:**
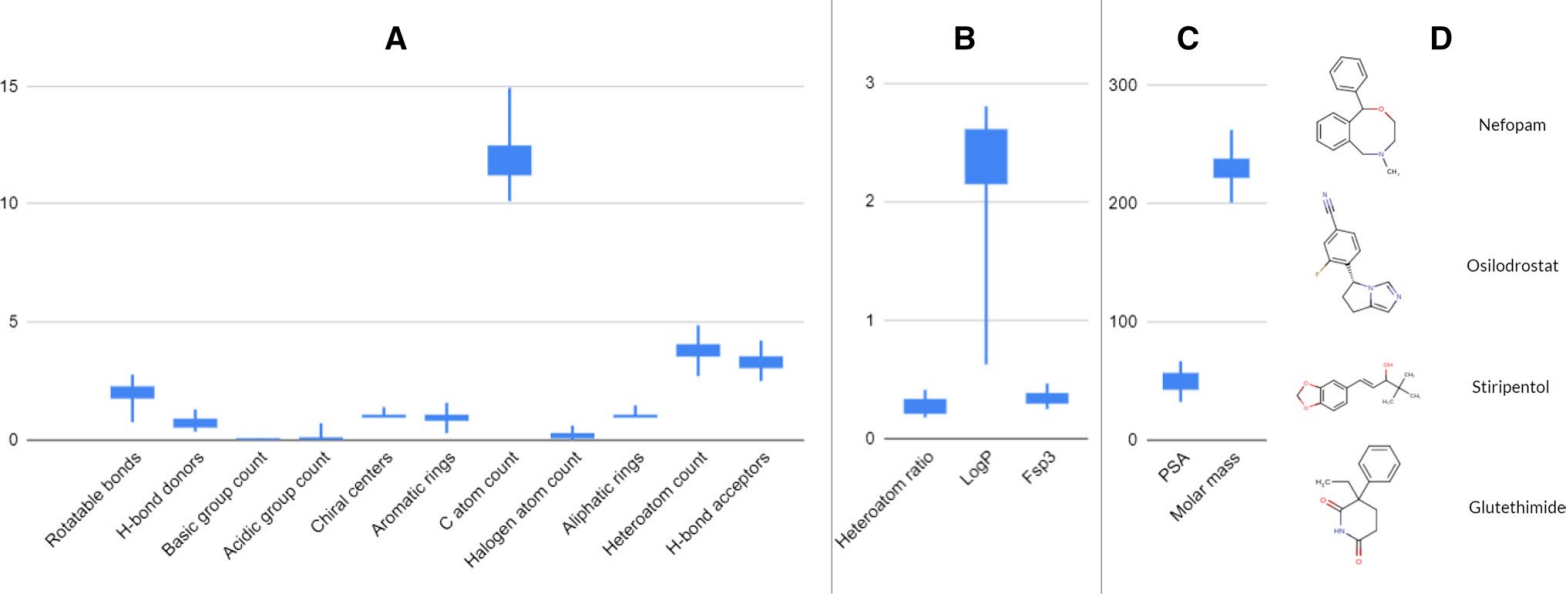
Property distributions of the grid points [35–45; 5–15] of the generated SOMs represented by 37 drugs (4 exemplified in Fig. 8d). Boxes cover the 2nd and 3rd quartile of the data, while lines cover 1st and 4th quartiles

Exemplary approved drugs residing in underrepresented property profile regions are shown in Fig. 6 and a more comprehensive overview of one of the largest underrepresented regions in Fig. 7.

**Fig. 8:**
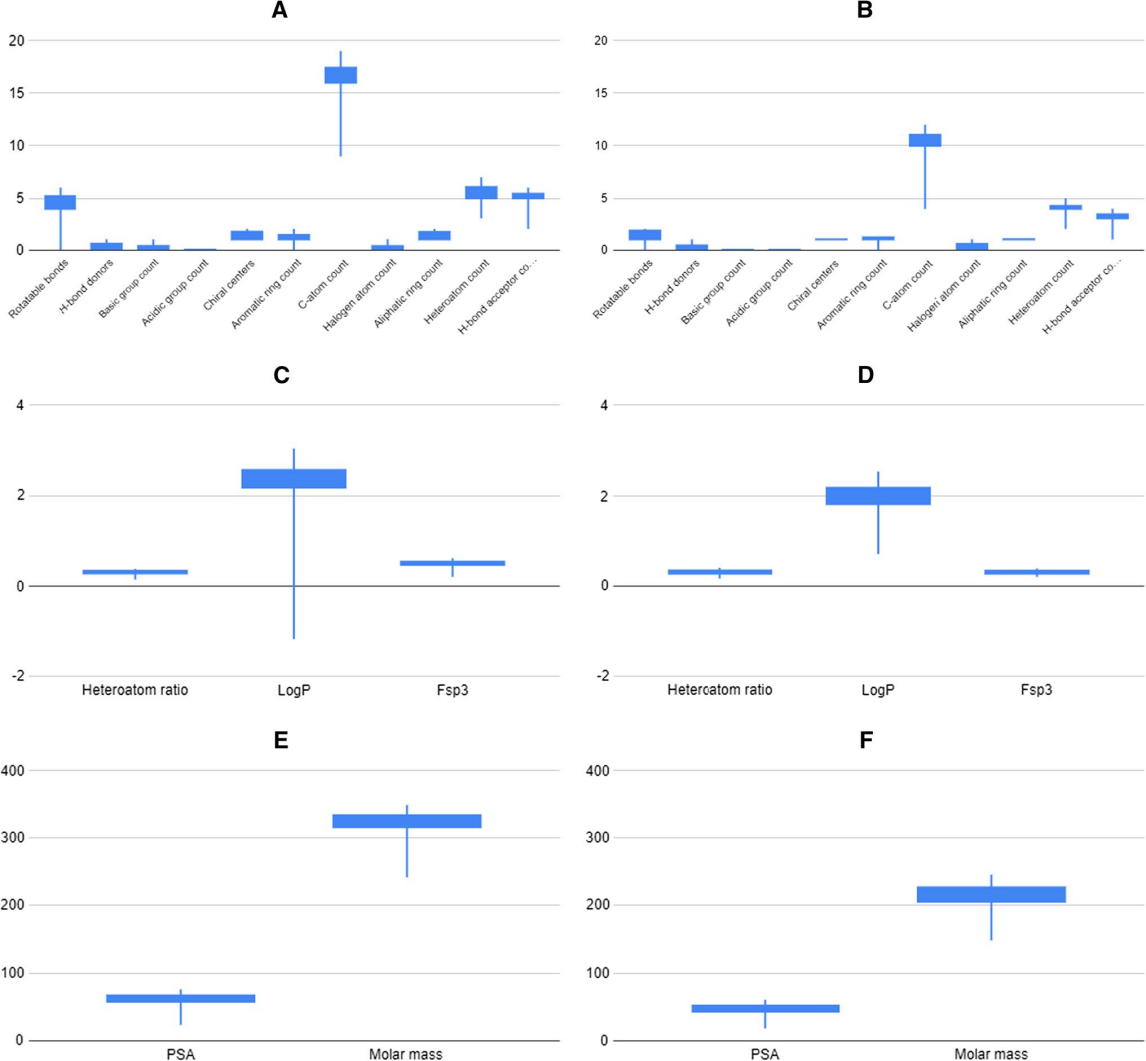
The property distribution of molecules residing on an overrepresented region [37:41];[20;24] is shown on the left (**a**, **c** and **e**) and the properties of molecules residing on a neighboring underrepresented region [38;42];[8;12] are shown on the right (**b**, **d** and **f**). Boxes cover the 2nd and 3rd quartile of the data, while lines cover 1st and 4th quartiles

We have selected an exemplary neighboring over- and an underrepresented region of Mcule stock database ([[37:41];[20;24]] and [[38;42];[8;12]], respectively) to analyze their physicochemical property profile differences in more detail. These regions have a similar distribution of some properties while they greatly differ in others— (Fig. 8). By comparing these two regions covering 16–16 gridpoints, we can see how these molecules differ from each other at the level of physicochemical properties. Notable differences can be seen in the number of rotatable bonds, carbon atoms and H-bond acceptors (Fig. 8a and b) (lower in case of the underrepresented molecules). PSA and molar mass (Fig. 8e and f) are lower too.

In contrast, halogen atom counts slightly different, while other properties such as heteroatom ratio (Fig. 3c and D) and H-bond donors are equivalent. In case of logP, we can see a moderate decrease in the over-versus to the underrepresented regions, but it is interesting to see the wide range in case of that property in the overrepresented regions. Comparing these distributions to the Ro5 filters, we can conclude that both examined regions comply with all the Ro5 rules and Ro5 were not able to distinguish them. The logP and molar mass distributions of the overrepresented set (left side) seem to fall a little closer to the Ro5 criteria: 500 vs. 330 and 5 versus 2.5, respectively. The biggest observed difference is in the number of H-bond donors but even this number in case of the overrepresented regions (~ 6) is far from the maximum (10) and the molecules identified in the underrepresented region have an even lower average value of 4.

## Conclusion

We analyzed the chemical space of approved drugs using self-organizing maps by means of sixteen widely used physicochemical parameters. The analysis of compounds described previously in drug discovery research programs (ChEMBL database) as well as compounds manufactured for drug discovery (individual supplier’s stock and virtual catalogs and the Mcule chemical marketplace database) revealed that there are still significant areas of the druglike property space that are yet unexplored. Furthermore, it has been shown that these regions represent compounds which are (i) compatible with very rigorous property filtering, (ii) not enriched in unwanted substructures, (iii) nor in potential promiscuous false positives. The presence of such property profiles emphasizes the need for commercially or otherwise accessible libraries representing these yet-unexplored portions of chemical space. Furthermore, our results suggest that in case of a sufficient number of hits/leads, it makes a lot of sense to prioritize compounds by Ro5, our described Strict filter or similar rules as there is still a lot to discover within such filtered space. On the other hands, in case the number of hits/leads are limited, it is suggested to investigate compounds beyond Ro5 as well [54], but one should be prepared for a longer development time and a higher risk of failure. Our presented approach based on self-organizing maps (SOMs) is well suited for the multiparameter analysis of the physicochemical parameter space and can identify property profiles that are otherwise missed by traditional filters such as Ro5 or Ro3 and they can thereby minimize false negatives and false positives of in silico ADMET screening. It has to be mentioned that the chemical space can be represented in an infinite number of ways, and in our study, we analyzed compounds from one aspect only: by sixteen physicochemical parameters. Nevertheless, these included descriptors most frequently applied by the industrial drug research programs. The results suggest that a virtual library based on the combination of an extensive and diverse set of stock building blocks and robust reactions can result in better sampling and could effectively populate the identified underrepresented regions. Such a library may be able to address difficult drug discovery targets associated with yet-unmet medical needs.

## Experimental section

### Databases

The supplier databases have been downloaded in either SMILES or SDF format. DrugBank (version: 5.0.10.) was downloaded in SDF format. For ChEMBL database, we used version 23 and where applicable we filtered it by the activity notations (pChEMBL > 6 and the activity comment is “active”). The records have been converted to SMILES, and structures were ionized at their physiological (pH = 7.4) state using Indigo toolkits. Mcule stock and supplier catalogs were downloaded and extracted from Mcule website (03/11/2020 https://mcule.com/database/).

### Filters

The Ro5 filters applied in our study were identical with the ones in the original publication [1] (Table S1). Our Strict filter was defined based on our industrial pharma partners’ feedback—see Table S1. For the elimination of the promiscuous and problematic substructures—as we called SMARTS filtering we used both Open Babel [55] and Indigo [56] and eventually applied the stricter of the two as we have noticed slight differences in their output. Furthermore, we used Lilly Medchem Rules as a standalone application for the most accurate elimination of such substructures. We have also contributed to the application with the addition of the feature to process files containing multiple SMARTS patterns. Settings were as follows:
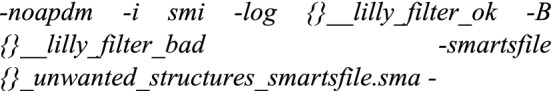


For the generation of the physicochemical properties, the SDF input files were applied. For those properties available in Open Babel (halogen atom count, heteroatom count, chiral center count, rotatable bonds, H-bond donors, H-bond acceptors, aromatic rings, molar mass, logP, PSA, heteroatom ratio, aliphatic rings), we used the Open Babel functions using default parameters. For the remaining properties (acidic group count; basic group count; carbon atom count, fsp3), we created our own SMARTS definitions and counted the number of matching atoms for each molecule:
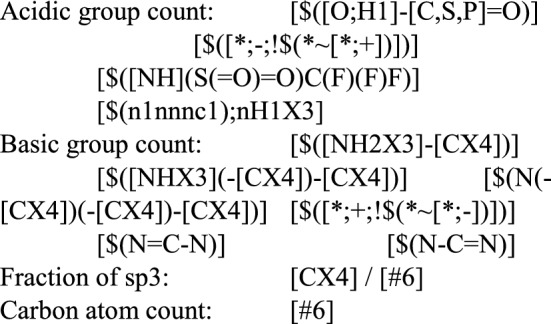


The generated SDF files have been converted to CSV and LRN files. (The latter was used as the input for generating the self-organized maps by Somoclu.)

### Self-organized maps (SOMs)

To generate self-organized maps, we applied Somoclu [57], implementation. Before the training of our self-organized maps, we had to collect the highest and lowest values for each property present in our initial sets that was dependent of the filtering methods used. For example, without property filtering, we had an extremely high number of chiral centers and molar mass values in certain datasets. Using these maximum and minimum values, we have normalized each property to a range of 0–1. Using the Somoclu implementation of a self-organizing map algorithm, we have created a map of 60 × 45 grid points—each of them had 16 dimensions. The size of 60 × 45 was sufficiently small to be trainable with our training datasets and the “deployment” of the big datasets could be achieved within a few hours on a standard computer (4 core CPU: i5-8250U; 8 GB RAM). The 60 × 45 mapsize was also sufficiently high for detailed comparison of the databases. Furthermore, choosing a not symmetrical map is beneficial for the visual evaluation and comparison.

During training, the 16 dimensions (properties) have been first filled with random values between 0 and 1. For the training of the maps, we have used the appropriate (property filtered) version of the DrugBank database. During the training process, each molecule of the training set was introduced to the map and the most similar grid point (using Euclidean distance calculation) was chosen as the winning node. This node and its neighbors were then modified to be more similar to the molecule introduced to them. This process was repeated for every molecule of the dataset in every epoch. In every following epoch, both the learning rate and the learning neighborhood area were lowered. The SOMs used in our study were trained for 500 epochs.

As a result, we got a map where the drugs—filtered by Strict filter—were evenly distributed. On these maps, we examined the distribution of the collected compound libraries (ChEMBL, Mcule stock, Supplier #1 stock, Supplier #1 virtual and Supplier #2-#7 catalogs). Each molecule of the catalogs was introduced to every gridpoint of the map and was assigned to the one with the lowest Euclidian distance (calculated between the 16 parameters of the molecules and the gripdpoints). Based on the number of molecules assigned to each gridpoint over- and underrepresented regions of the commercial and non-commercial databases were identified. Underrepresented gridpoints were defined as follows: number of molecules on gridpoint < (total number of molecules/2700/5). Overrepresented gridpoints were defined as follows: number of molecules on gridpoint > (all molecules/2700 * 5). Furthermore, we calculated map heterogeneity scores to characterize the heterogeneity of SOMs’ physicochemical property distributions as follows: ((number of overrepresented gridpoints + number of underrepresented gridpoints)/total number of gridpoints), thus the lower the map heterogeneity score, the higher the diversity of the database.

The heatmaps and Figure S1 were created by using matplotlib [58] functions.

## Supplementary Information

Below is the link to the electronic supplementary material.

Supplementary file1 (PDF 889 kb)
