# Supplementary material for: Analysis of the uncharted, druglike property space by self-organizing maps
Source: Mol Divers. 2021 Oct 28;26(5):2427–41. doi: 10.1007/s11030-021-10343-y (PMC9532340; doi:10.1007/s11030-021-10343-y)
Supplement: Supplementary file 1 — Supplementary file1 (PDF 889 kb) [file 11030_2021_10343_MOESM1_ESM.pdf]

SUPPORTING INFORMATION

Rule of five (Ro5)		
Molar mass	-	500
LogP	-	5
H-bond donors	-	5
H-bond acceptors	-	10
Strict filter		
Physicochemical parameters	Minimum value	Maximum value
Rotatable bonds	0	10
H-bond donors	0	5
Heteroatom ratio	0.1	1.5
LogP	-2	3.5
Acidic group count	0	2
Chiral center	0	2
PSA	1	120
Fsp3	0.2	1
Aromatic rings	0	4
Molar mass	110	450
C atom count	3	27
Halogen atom count	0	6
Basic group count	0	2
Aliphatic rings	1	5
Hetero atom count	1	13
H-bond acceptors	1	10

Table S1. Criteria of *Rule of five* and our filter set (referred as *Strict filter* in the manuscript) built based on the feedback of pharmaceutical companies.

	DrugBank	ChEMBL	Mcule stock	Supplier #1 stock	Supplier #1 virtual
Strict filters					
Input count	8,646	1,727,112	9,169,152	3,397,680	336,985,480
Output count	836 (10%)	242,768 (14%)	2,669,910 (29%)	1,160,797 (34%)	115,096,630 (34%)
Aliphatic rings max	81 (0.9%)	14,899 (0.9%)	2,433 (0.0%)	97 (0.0%)	45,025 (0.0%)
Aliphatic rings min	4,081 (14%)	679,747 (39%)	3,611,387 (39%)	1,339,943 (39%)	107,107,947 (32%)
LogP max	3,177 (37%)	1,011,783 (59%)	4,050,255 (44%)	1,194,318 (35%)	134,313,306 (40%)
LogP min	471 (5.4%)	9,080 (0.5%)	5,166 (0.1%)	424 (0.0%)	5,651 (0.0%)
Fsp3 min	1,891 (22%)	448,543 (26%)	2,066,297 (23%)	598,325 (18%)	28,491,552 (8.5%)
PSA max	2,515 (29%)	362,170 (21%)	878,774 (10%)	259,739 (7.6%)	25,369,739 (7.5%)
PSA min	72 (0.8%)	2,206 (0.1%)	16,446 (0.2%)	8,181 (0.2%)	187 (0.0%)
Molar mass max	1,822 (21%)	520,597 (30%)	1,213,989 (13%)	248,928 (7.3%)	7,942,355 (2.4%)
Molar mass min	257 (3.0%)	1,263 (0.1%)	6,278 (0.1%)	4,254 (0.1%)	168 (0.0%)
Chiral centers max	2,217 (26%)	271,944 (16%)	354,926 (3.9%)	114,052 (3.4%)	22,210,388 (6.6%)
Basic group count max	347 (4.0%)	94,934 (5.5%)	164,014 (1.8%)	42,193 (1.2%)	9,541,186 (2.8%)
Heteroatom ratio max	233 (2.7%)	2,271 (0.1%)	3,930 (0.0%)	1,708 (0.1%)	1,318 (0.0%)
Heteroatom ratio min	331 (3.8%)	30,263 (1.8%)	98,411 (1.1%)	42,111 (1.2%)	236,154 (0.1%)
C atom count max	880 (10%)	276,743 (16%)	325,415 (3.5%)	31,057 (0.9%)	375,534 (0.1%)
C atom count min	187 (2.2%)	816 (0.0%)	1,513 (0.0%)	781 (0.0%)	10 (0.0%)
H-bond acceptors max	1,191 (14%)	144,559 (8.4%)	129,282 (1.4%)	19,444 (0.6%)	3,778,676 (1.1%)
H-bond acceptors min	79 (0.9%)	2,012 (0.1%)	16,815 (0.2%)	7,602 (0.2%)	3,031 (0.0%)
H-bond donors max	808 (9.3%)	71,528 (4.1%)	9,454 (0.1%)	231 (0.0%)	0 (0.0%)
Aromatic rings max	178 (2.1%)	78,143 (4.5%)	151,353 (1.7%)	15,387 (0.5%)	653,512 (0.2%)
Heteroatom count max	779 (9.0%)	101,048 (5.9%)	34,308 (0.4%)	1,441 (0.0%)	130,884 (0.0%)
Heteroatom count min	9 (0.1%)	490 (0.0%)	2,262 (0.0%)	747 (0.0%)	178 (0.0%)
Halogen atom count max	27 (0.3%)	5,671 (0.3%)	9,557 (0.1%)	1,352 (0.0%)	143,350 (0.0%)
Acidic group count max	705 (8.2%)	23,072 (1.3%)	7,104 (0.1%)	1,004 (0.0%)	35,073 (0.0%)
Rotatable bonds max	1,228 (14%)	247,764 (14%)	338,271 (3.7%)	111,715 (3.3%)	18,845,511 (5.6%)
Ro5					
0 violation	6,269 (73%)	1,132,839 (66%)	7,819,921 (85%)	3,179,689 (94%)	317,662,607 (94%)
1 violation	1,129 (13%)	326,628 (19%)	1,065,200 (12%)	205,941 (6.1%)	19,195,555 (5.7%)

2 violations	643 (7.4%)	181,207 (10%)	257,435 (2.8%)	11,558 (0.3%)	127,318 (0.0%)
3 violations	536 (6.2%)	69,195 (4.0%)	25,937 (0.3%)	479 (0.0%)	0 (0.0%)
4 violations	69 (0.8%)	16,779 (1.0%)	659 (0.0%)	13 (0.0%)	0 (0.0%)
Molar mass max	1,206 (14%)	321,324 (26%)	373,439 (4.1%)	15,391 (0.5%)	8 (0.0%)
H-bond acceptors max	1,191 (14%)	144,559 (8.4%)	129,282 (1.4%)	19,444 (0.6%)	3,778,676 (1.1%)
LogP max	1,094 (13%)	426,332 (25%)	1,148,342 (13%)	195,480 (5.8%)	15,671,507 (4.7%)
H-bond donors max	808 (9.3%)	71,528 (4.1%)	9,454 (0.1%)	231 (0.0%)	0 (0.0%)

Table S2. Size of the unfiltered and filtered databases by Ro5 and Strict filter and the number of molecules excluded by each rule individually.

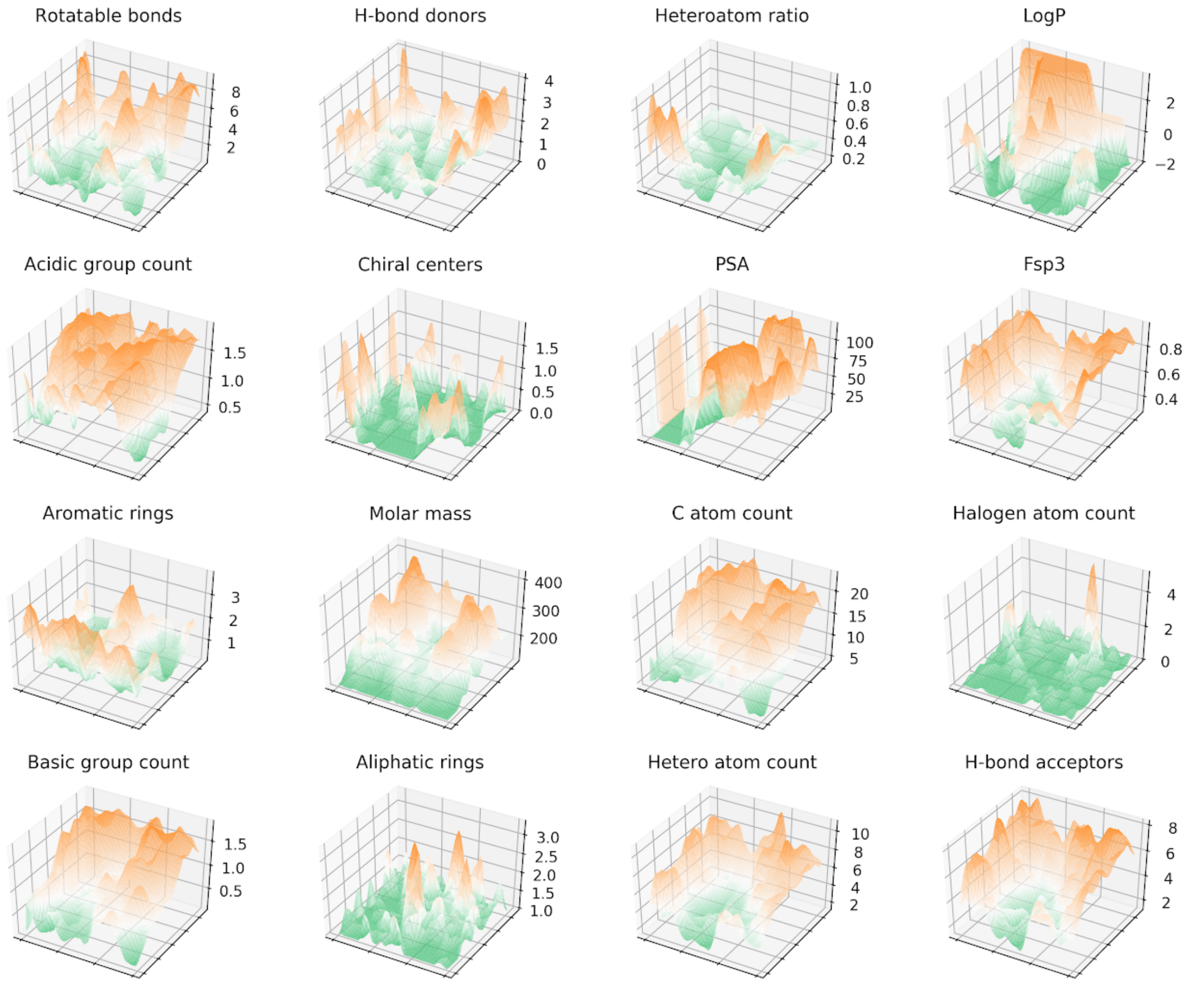

Figure S1. Distribution of the sixteen physicochemical properties on the SOM trained on 836 drugs. Where the X and Y axes are the coordinates over the trained map and the Z-axis shows the value of the given property at the given point. The ranges of the properties vary on each diagram (orange indicates higher and dark green indicates lower values of the given property).
